# Supplementary material for: Index cases first identified by nasal-swab rapid COVID-19 tests had more transmission to household contacts than cases identified by other test types
Source: PLoS One. 2023 Oct 5;18(10):e0292389. doi: 10.1371/journal.pone.0292389 (PMC10553276; doi:10.1371/journal.pone.0292389)
Supplement: S1 File — Containing S1 Table (Participant Demographics by [A] Study Phase and [B] Infecting SARS-CoV-2 Variant), S2 Table (Univariable Model), S3 Table (Association of Test Type Subcategories with SARS-CoV-2 Transmission Among Household Contacts), S4 Table (Results of Modeled Risk of Transmission to Household Contacts with Alternative Age Grouping), S5 Table (Results of Modeled Risk of Transmission to Household Contacts of Index Cases Infected with Omicron Variant), Supplemental Methods, and Supplemental References. (PDF) [file pone.0292389.s001.pdf]

Supplemental Information for:

Index Cases first identified by nasal swab rapid COVID-19 tests had more transmission to household contacts than Cases identified by other test types

Jenny Ji[1¶], Alexander Viloria Winnett BS[1,2¶], Natasha Shelby PhD[1], Jessica A. Reyes MPH[1], Noah W. Schlenker BS[1], Hannah Davich MPH[1], Saharai Caldera BS[1], Colten Tognazzini BSN[4], Ying-Ying Goh MD MPH[4], Matthew Feaster PhD MPH[4], Rustem F. Ismagilov PhD[1*]

[1]California Institute of Technology, Pasadena, CA, USA

[2]University of California Los Angeles – California Institute of Technology Medical Scientist Training Program, Los Angeles, CA USA

[3]Pasadena Public Health Department, Pasadena, CA, USA

¶ These authors contributed equally to this report.

*Correspondence to: rustem.admin@caltech.edu

Contents

S1 Table. Participant Demographics by (A) Study Phase and (B) Infecting SARS-CoV-2 Variant.

S2 Table. Univariable Model.

S3 Table. Association of Test Type Subcategories with SARS-CoV-2 Transmission Among Household Contacts.

S4 Table. Results of Modeled Risk of Transmission to Household Contacts with Alternative Age Grouping.

S5 Table. Results of Modeled Risk of Transmission to Household Contacts of Index Cases Infected with Omicron Variant.**Supplemental Methods**

Supplemental References

Detailed Author Contribution Statements

S1 Table. Participant Demographics by (A) Study Phase and (B) Infecting SARS-CoV-2 Variant. Demographics, vaccination status, and index case testing type of the 85-household cohort divided by (**A**) study phase and (**B**) infecting viral variant.

		(A) Study Phase				(B) Viral Variant			
		Phase I		Phase II		Omicron		Ancestral Variants	
		N= 155		N= 215		N= 163		N= 207	
Self-Reported Gender Identity*		n	(%)	n	(%)	n	(%)	n	(%)
	Man	70	45.2	104	48.4	78	47.9	96	46.4
	Woman	85	54.8	111	51.6	85	52.1	111	53.6
	Third Gender	0	0.0	0	0.0	0	0.0	0	0.0
Age Category		n	(%)	n	(%)	n	(%)	n	(%)
	<10	29	18.7	28	13.0	20	12.3	37	17.9
	10 to 60	115	74.2	173	80.5	129	79.1	159	76.8
	>60	11	7.1	14	6.5	14	8.6	11	5.3
Self-Reported Race/Ethnicity**		n	(%)	n	(%)	n	(%)	n	(%)
	Asian or Pacific Islander	11	7.1	36	16.7	31	19.0	16	7.7
	Biracial	8	5.2	8	3.7	6	3.7	10	4.8
	Black/African American	9	5.8	7	3.3	7	4.3	9	4.3
	Native American/Alaska	0	0.0	12	5.6	8	4.9	4	1.9
	Unknown	61	39.4	66	30.7	44	27.0	83	40.1
	White, Hispanic	38	24.5	30	14.0	22	13.5	46	22.2
	White, Non-Hispanic	28	18.1	56	26.0	45	27.6	39	18.8
Vaccination Status***		n	(%)	n	(%)	n	(%)	n	(%)
	Unvaccinated	133	85.8	15	7.0	4	2.5	144	69.6
	Partial	3	1.9	2	0.9	2	1.2	3	1.4
	Complete	1	0.6	77	35.8	51	31.3	27	13.0
	Boosted	0	0.0	79	36.7	72	44.2	7	3.4
	Unknown	18	11.6	42	19.5	34	20.9	26	12.6
Household Size		n	(%)	n	(%)	n	(%)	n	(%)
	<= 4	97	62.6	86	40.0	66	40.5	117	56.5
	> 4	58	37.4	129	60.0	97	59.5	90	43.5
Index Case Test Type		n	(%)	n	(%)	n	(%)	n	(%)
	Rapid Nasal	20	12.9	64	29.8	54	33.1	30	14.5
	Not Rapid Nasal	131	84.5	110	51.2	77	47.2	164	79.2
	Unknown	4	2.6	41	19.1	32	19.6	13	6.3

*Both sex assigned at birth and current gender identity were self-reported by participants. One participant reported male assignment at birth and current gender identity of woman. Reported gender is listed.

**63 individuals currently listed as 'Unknown' did not select a race category but wrote-in "Latino"/"Latina"/"Latinx."

***Participants reported date and manufacturer of each vaccine dose received; vaccination status was defined only by doses received at least 7 days prior to enrollment in the study. Unvaccinated was defined as having received no COVID-19 vaccine doses. Partial vaccination was defined as receiving one dose of a multiple-dose series (e.g., Pfizer-BioNTech, Moderna). Complete vaccination was defined as receiving all doses of an initial COVID-19 vaccine series. Boosted was defined as the participant receiving any dose beyond an initial COVID-19 vaccine series.

S2 Table. Univariable Model. Simple Odds Ratios (OR) for covariables included in the models in Fig 2.

	Univariable Model					
**Exposure**	**SAR (%)**	**95% CI (%)**	**Pairwise Comparison Test**	**OR**	**95% CI**	**Wald Test**
**Age Category**						
<10	41.4	23.5-59.3		1.22	0.55-2.70	P=0.63
10 to 60	36.7	29.8-43.6		1.00	*(Reference)*	
>60	7.1	0.0-20.6	NA	0.13	0.02-1.04	P=0.05
**Race/Ethnicity**						
Asian/PI	30.3	14.6-46.0		0.91	0.37-2.25	P=0.84
Biracial	33.3	6.66-60.0		1.05	0.28-3.87	P=0.94
Black	21.4	0.0-42.9		0.57	0.14-2.27	P=0.43
Native American	12.5	0.0-35.4		0.30	0.03-2.59	P=0.27
Unknown	43.4	30.1-56.7		1.61	0.76-3.41	P=0.22
White Hispanic	43.5	29.2-57.8		1.61	0.74-3.52	P=0.23
White Non-Hispanic	32.3	20.9-43.7	NA	1.00	*(Reference)*	
**Viral Variant**						
Ancestral	34.6	26.4-42.8		1.00	*(Reference)*	
Omicron	36.6	27.2-46.0	P=0.75	1.09	0.63-1.88	P=0.75
**Vaccination Status**						
Not Vaccinated	38.5	29.1-47.8		1.00	*(Reference)*	
Vaccinated*	33.1	24.9-41.3	P=0.40	0.79	0.46-1.36	P=0.40
**Household Density**						
≤4	24.1	16.2-32.0		1.00	*(Reference)*	
>4	46.2	37.3-55.2	**P<0.001**	2.71	1.54-4.75	**P=0.001**
**Infection Control Practice****						
Not sharing food	39.0	29.4-48.6	P=0.53	1.19	0.69-2.05	P=0.53
Not eating meals together	30.8	23.2-38.3	**P=0.01**	0.49	0.28-0.86	**P=0.01**
Not sharing a bedroom	30.2	23.4-37.1	**P<0.001**	0.30	0.16-0.58	**P<0.001**
Not sharing a bathroom	26.8	18.0-35.6	**P=0.005**	0.46	0.26-0.81	**P=0.007**
Not share personal items	33.8	25.9-41.7	P=0.24	0.72	0.41-1.25	P=0.24
Avoid contact with clothes	34.5	26.7-42.2	P=0.34	0.76	0.43-1.33	P=0.33
Social distancing	31.2	23.1-39.3	P=0.05	0.58	0.33-1.01	P=0.05
Mask use	32.3	24.4-40.3	P=0.10	0.62	0.36-1.09	P=0.10
**Index Case Diagnostic Test**						
Rapid Nasal Test	50.9	37.9-63.9		2.61	1.39-4.91	**P=0.003**
Other Test Type	28.4	21.1-35.6	**P=0.003**	1.00	*(Reference)*	

S3 Table. Association of Test Type Subcategories with SARS-CoV-2 Transmission Among Household Contacts. Provides data and Odds Ratios (OR) on the association between COVID-19 test type used to identify the household index case and subsequent transmission to household contacts.

	Uninfected		Secondary Case		Multivariable Model					
**Exposure**	**N**	**(%)**	**N**	**(%)**	**aSAR**	**95% CI**	**aOR**	**95% CI**	**Wald Test**	**Likelihood Ratio Test**
**Test Type**	**149**		**82**							
Nasal Rapid	28	18.8	31	37.8	48.1	32.6-63.5	1.00	*(Reference)*		**P=0.05**
Nasal Not Rapid	37	24.8	7	8.5	16.2	4.59-27.8	0.13	0.03-0.51	**P=0.003**	
Nasopharyngeal Rapid	17	11.4	4	4.9	19.5	0.25-38.7	0.17	0.03-1.06	P=0.06	
Nasopharyngeal Not Rapid	31	20.8	15	18.3	33.1	18.0-48.2	0.43	0.11-1.61	P=0.21	
Oral Rapid	1	0.7	3	3.7	43.9	0.0-98.4	0.80	0.04-18.01	P=0.89	
Oral Not Rapid	17	11.4	10	12.2	43.4	20.7-66.1	0.78	0.15-3.88	P=0.76	
Unknown	18	12.1	12	14.6	45.9	26.9-64.9	0.89	0.24-3.28	P=0.86	

S4 Table. Results of Modeled Risk of Transmission to Household Contacts with Alternative Age Grouping. Counts (N) of enrolled individuals who did not become infected during enrollment (uninfected) or became infected after the index case (secondary case) are provided for each alternative age category. The adjusted secondary attack rate (aSAR) and adjusted odds ratio (aOR) point estimates with 95% confidence intervals from multivariable analysis are listed for each covariable. The same analysis in **Fig. 2A** is performed with alternative age stratification.

	Uninfected		Secondary Case		Multivariable Model						
**Exposure**	**N**	**(%)**	**N**	**(%)**	**aSAR**	**95% CI**	**Pairwise Comparison Test**	**aOR**	**95% CI**	**Wald Test**	**Likelihood Ratio Test**
**Age Category**	**149**		**82**								
<12	24	16.1	16	19.5	40.0	24.7-55.4	NA	0.95	0.36-2.53	P=0.92	P=0.06
12 to 18	26	17.4	12	14.6	31.8	17.6-46.0		0.75	0.27-2.07	P=0.58	
19 to 60	86	57.7	53	64.6	37.4	29.0-45.9		1.00	*(Reference)*		
>60	13	8.7	1	1.2	9.8	0.0-26.9c		0.07	0.01-0.74	**P=0.03**	
**Index Case Diagnostic Test**	**134**		**71**								
Rapid Nasal Test	28	20.9	29	40.8	53.7	35.1-68.5	**P=0.003**	4.92	1.67-14.50	**P=0.004**	P=0.07
Other Test Type	106	79.1	42	59.2	27.1	19.4-34.8		1.00	*(Reference)*		

S5 Table. Results of Modeled Risk of Transmission to Household Contacts of Index Cases Infected with Omicron Variant. Among 101 household contacts of index cases infected with the Omicron variant, counts (N) of enrolled individuals who did not become infected during enrollment (uninfected) or became infected after the index case (secondary case) are provided for each covariable. The adjusted secondary attack rate (aSAR) and adjusted odds ratio (aOR) point estimates with 95% confidence intervals from multivariable analysis are listed for each covariable. As all households were infected with the Omicron variant, viral variant was removed from the sufficient set of covariables (**Figure 1C**). Of note, for 21 household contacts the index case test type was unknown and excluded from analysis. Additionally, two of two household contacts who were unvaccinated were both infected, resulting in collinearity of the vaccination covariable with infection status and omission of these two individuals. Similarly, six of six household contacts over the age of 60 were uninfected, resulting in collinearity of this age category with infection status and omission of these six individuals. Further, five of five individuals self-identifying as Native American were uninfected, resulting in collinearity of this race category with infection status and omission of these five individuals. A total of 67 household contacts were analyzed in this multivariable model. **(A)** Data for four covariables. **(B)** Association between COVID-19 test type used to identify the household index case, and subsequent transmission to household contacts when controlling for covariables. *Vaccinated status is defined as having received at least one dose of a COVID-19 vaccine at least 7 days prior to enrollment.

(A)

	Uninfected		Secondary Case		Multivariable Model					
**Exposure**	**N**	**(%)**	**N**	**(%)**	**aSAR**	**95% CI**	**Pairwise Comparison Test**	**aOR**	**95% CI**	**Wald Test**
**Age Category**	**64**		**37**							
<10	4	6.3	3	8.1	50.4	14.6-86.2		1.89	0.09-41.59	P=0.09
10 to 60	54	84.4	34	91.9	37.4	24.4-50.3	NA	1.00	*(Reference)*	
>60	6	9.4	0	0.0						
**Race/Ethnicity**	**64**		**37**							
Asian/PI	15	23.4	9	24.3	38.3	14.1-62.5		1.37	0.15-12.44	P=0.78
Biracial	3	4.7	3	8.1	36.5	1.1-71.9		1.19	0.06-22.77	P=0.91
Black	4	6.3	3	8.1	66.1	26.5-100.0		5.71	0.15-214.26	P=0.35
Native American	5	7.8	0	0.0			NA			
Unknown	4	6.3	4	10.8	66.6	29.0-100.0		11.59	0.36-389.02	P=0.17
White Hispanic	10	15.6	5	13.5	44.3	0.0-46.9		0.29	0.02-5.00	P=0.39
White Non-Hispanic	23	35.9	13	35.1	33.0	14.5-51.6		1.00	*(Reference)*	
**Vaccination Status**	**64**		**37**							
Not Vaccinated	0	0.0	2	5.4			NA	1.00	*(Reference)*	
Vaccinated*	64	100.0	35	94.6	38.1	25.3-50.9				
**Household Size**	**64**		**37**							
≤4	31	48.4	13	35.1	28.6	11.9-45.4	P=0.19	1.00	*(Reference)*	
>4	33	51.6	24	64.9	45.7	26.5-64.9		3.82	0.49-29.62	P=0.20

(B)

**Index Case Diagnostic Test**	**52**		**28**							
Rapid Nasal Test	34	65.4	8	28.6	55.6	35.2-76.1	**P=0.003**	15.89	1.59-158.41	**P=0.02**
Other Test Type	18	34.6	20	71.4	16.7	2.8-30.5		1.00	*(Reference)*	

Supplemental Methods

Participants

Individuals fluent in English or Spanish aged 6 years and older from households of two or more persons were eligible for participation if at least one household member had tested positive, developed COVID-19-like symptoms,[1] or had a known exposure with a SARS-CoV-2 infected individual within 7 days, and at least one other household member had either negative or unknown infection status during screening.

Upon enrollment, participants completed a questionnaire to provide information about demographics (based on the 2019 California Health Interview Survey tool)[2], medical information, and COVID-19 history (e.g., COVID-19-like symptoms[1], positive and negative test results, and COVID-19 vaccination information). For participants enrolled prior to February 22, 2021, vaccination was not asked, but unvaccinated status was inferred based on local vaccine availability.[3] Vaccination status was defined only by doses received at least 7 days prior to enrollment. The questionnaire also asked about household size, the age and gender of other household members and their SARS-CoV-2 infection status, as well as current and anticipated infection-control practices (e.g., shared items and spaces, disinfection, distancing, and masking).

Sample collection

In Phase I of the study, participants self-collected either saliva or paired saliva and anterior-nares nasal swabs every morning upon waking and in the evening before bed in Spectrum SDNA 1000 devices.[4] In Phase II, participants self-collected paired saliva, anterior-nares nasal swabs, and oropharyngeal swabs in Zymo Research's SafeCollect devices[5,6] once daily (upon enrollment and thereafter each morning upon waking).

Supplemental References

1. CDC. Symptoms of Coronavirus. 2020:https://www.cdc.gov/coronavirus/2019-ncov/symptoms-testing/symptoms.html.
2. UCLA. California Health Interview Survey: 2019 Adult CAWI Questionnaire 2019.
3. LA County Public Health. Los Angeles County Public Health COVID-19 Vaccine Dashboard. 2022; http://publichealth.lacounty.gov/media/coronavirus/vaccine/vaccine-dashboard.htm.
4. FDA. Emergency use authorization (EUA) review memorandum sdna-1000 saliva collection device. 2021; https://www.fda.gov/media/142907/download.
5. Zymo Research. SafeCollect Saliva Collection Kit User Instruction Manual. 2021; https://files.zymoresearch.com/protocols/r1211e-dna_rna_shield_safecollect_saliva_collection_kit_user_instructions.pdf.
6. Zymo Research. SafeCollect Swab Collection Kit User Instruction Manual. 2021; https://files.zymoresearch.com/protocols/r1160_r1161-dna_rna_shield_safecollect_swab_collection_kit_user_Instructions.pdf.

Detailed Author Contribution Statements

Listed alphabetically by surname

Saharai Caldera (SC)- Study coordinator; recruited, enrolled and maintained study participants with NS, JAR, HD and NWS; study-data quality control; validated data provided by participants in study instruments.

Hannah Davich (HD)- Lead study coordinator; recruited, enrolled and maintained study participants with NS, JAR, SC and NWS; developed recruitment strategies and did outreach with NS; study-data quality control; validated data provided by participants in study instruments; Compiled data from screening with NS for use in Fig 1B. Compiled data for household size and density metrics.

Matthew Feaster (MF)- Co-investigator; contributed to overall study design and recruitment strategies; provided guidance and expertise on SARS-CoV-2 epidemiology and local trends. Contributed to design of biostatistical analysis, particularly conceptualization of causal model represented in Fig 1C and parameterization/coding of covariables. Technical guidance on analysis method and interpretation. Reviewed manuscript. AC

Ying-Ying Goh (YYG)- Co-investigator; contributed to overall study design and recruitment strategies; provided guidance and expertise on SARS-CoV-2 epidemiology and local trends.

Rustem F. Ismagilov (RFI)- Principal investigator; provided leadership, technical guidance, oversight of all analyses, and was responsible for obtaining the primary funding for the study.

Jenny Ji (JJ)- Conceptualization of study with AVW and RFI. Performed extensive literature search on household transmission and co-wrote enrollment questionnaire with AW and NS. Data curation. Performed preliminary analyses. Coded and cleaned data from participant questionnaires. Validated underlying data. Parameterization of participant data for analysis. Assigned index case with AVW, NS, and NWS. Performed analyses in STATA. Prepared Figure 1, 2 and 3 with AVW. Prepared Table 1, Table S1, Table S2, and Table S3 with AVW. Prepared Tables S4 and S5. Study-specific literature review with AVW. Outlined manuscript with AVW. Revised manuscript with AVW.

Jessica A. Reyes (JAR)- Lead study coordinator; recruited, enrolled and maintained study participants with NS, HD, SC, and NWS; study-data quality control; validated data provided by participants in study instruments.

Natasha Shelby (NS)- Study administrator; contributed to initial study design and recruitment strategies; co-wrote enrollment questionnaire with AW and JJ; hired, trained, and supervised the study-coordinator team; recruited, enrolled and maintained study participants with JAR, JAR, NWS, HD and SC; study-data quality control; validated data provided by participants in study instruments; data curation; organized archiving of participant data; helped assemble CONSORT diagram (Fig 1B); assisted with assignment of household index cases with JJ, AVW, and NWS; managed reference library; reviewed and edited the manuscript.

Noah W. Schlenker (NWS)- Study coordinator; recruited, enrolled and maintained study participants with NS, JAR, HD and SC; study-data quality control; validated data provided by participants in study instruments; major role in assignment of household index cases with JJ, AVW, and NS.

Colten Tognazzini (CT)- Coordinated the recruitment efforts at PPHD with case investigators and contact tracers; provided guidance and expertise on SARS-CoV-2 epidemiology and local trends.

Alexander Viloria Winnett (AVW)- Conceptualization of study with JJ and RFI. Contributed to overall study design and recruitment strategies. Co-wrote enrollment questionnaire with NS and JJ; Data curation and analysis. Assigned vaccination status for each participant. Assigned infection status from viral load data. Assigned index case with JJ, NS, and NWS. Prepared Figure 1, 2 and 3 with JJ. Prepared Table 1, Table S1, Table S2, and Table S3 with JJ. Verified underlying data and analyses performed by JJ. Study-specific literature review with JJ. Outlined manuscript with JJ. Drafted initial manuscript. Revised manuscript with JJ.

Contact information and ORCiDs:

Jenny JI, jji@caltech.edu, 626-395-3464

ORCID: 0000-0002-7901-5605

Alexander VILORIA WINNETT, awinnett@caltech.edu, 626-395-3464

ORCID: 0000-0002-7338-5605

Natasha SHELBY, tshelby@caltech.edu, 626-395-3464

ORCID: 0000-0001-9097-3663

Jessica A. REYES, jayrey628@gmail.com, 626-395-3464

ORCID: 0000-0002-5507-7633

Noah W. SCHLENKER, noahschlenker@gmail.com, 626-395-3464

ORCID: 0000-0002-8581-4403

Hannah DAVICH, hannahdavich@gmail.com, 626-395-3464

ORCID: 0000-0001-6880-3086

Saharai CALDERA, calderasaharai@gmail.com. 626-395-3464

ORCID: 0000-0001-5057-9186

Colten TOGNAZZINI, ctognazzini@cityofpasadena.net, 626-744-6000

ORCID: 0000-0002-2754-3588

Ying-Ying GOH, ygoh@cityofpasadena.net, 626-744-6000

ORCID: 0000-0001-5136-7214

Matthew FEASTER, mfeaster@cityofpasadena.net, 626-744-6000

ORCID: 0000-0001-9966-2845

*Corresponding author: Rustem F. ISMAGILOV, rustem.admin@caltech.edu, 626-395-8130

ORCID: 0000-0002-3680-4399
