# Supplementary material for: Index cases first identified by nasal-swab rapid COVID-19 tests had more transmission to household contacts than cases identified by other test types
Source: PLoS One. 2023 Oct 5;18(10):e0292389. doi: 10.1371/journal.pone.0292389 (PMC10553276; doi:10.1371/journal.pone.0292389)
Supplement: Attachment — Submitted filename: Journal_Reviewer_Response_PLOS-One_update.pdf [file pone.0292389.s002.pdf]

Point-By-Point Response

Referee: 1

Although COVID-19 infection currently is not a public health emergency, the lessons learnt can be used for future pandemic. The deep understanding of COVID-19 infection will support the response to other respiratory virus. Household transmission is a challenge to control the infection. The authors presented a relevant study to the understand the role of rapid tests to control the transmission of COVID-19 among the household members.

We thank the Reviewer for their review of our manuscript, and recognition of the importance of household transmission to control spread of not only SARS-CoV-2 but other respiratory viruses.

1. Delta and Omicron variants were concern to public health systems due to high transmission rate. Why the authors only conducted the analysis for Omicron variant. I recommend to do the same for Delta variant.

We thank the Reviewer for this comment, which we agree would enhance the paper. However, since there are only 12 index cases that tested positive with the Delta variant and 48 total participants exposed to the variant, this analysis would be underpowered for these analyses.

2. Several rapid tests, with different specificity and sensitivity, were developed for COVID-19 diagnosis. Assuming the variation on diagnostic performance, which rapid test was considered for this study? The test sensitivity is not a confounding?

We appreciate this point about test sensitivity raised by this reviewer. We agree that COVID-19 tests exhibit a wide range of analytical sensitivities, with limits of detection spanning from as low as 100 copies/mL of specimen to 100,000,000 copies/mL of specimen. In order to provide fast turnaround times, rapid COVID-19 tests typically forgo specimen processing steps, such as purification or concentration of viral materials that increase analytical sensitivity. In our analysis, we investigate the effect of differences in analytical sensitivity (and turnaround time) by stratifying the analysis of test type into nasal rapid COVID-19 tests which we assume to have low analytical sensitivity, and other test types that would presumably have high analytical sensitivity.

We do not know which exact tests were taken by our participants, because the initial testing took place prior to their enrollment in the study. As an observational study, participants likely took several different commercially available rapid COVID-19 tests, rather than a single brand of rapid COVID-19 test. But because rapid COVID-19 tests exhibit similar, low analytical sensitivity, we believe it is appropriate to describe them as a type of COVID-19 test (as opposed to high analytical sensitivity COVID-19 tests). We describe this point in lines 140-146:

> Participants were not asked to report the specific test name, laboratory platform, or viral target (e.g., molecular, antigen), due to concerns that laypersons would not be aware of these terms (especially if the test was run by a clinic rather than direct-to-consumer). However, rapid tests (both antigen and molecular) have characteristically low analytical sensitivity because they forego the time-consuming and technically challenging extraction steps to purify and concentrate viral targets. Because our hypothesis was related to low-analytical-sensitivity rapid tests performed on specimens from nasal swabs, we simply distinguish rapid tests from those with longer turnaround times and presumably higher analytical sensitivity (**Fig 1D**).

The point by the Reviewer is well-taken, as there is always a risk that participants will incorrectly report the type of COVID-19 test taken in the community prior to enrollment in the study. However, we expect misreporting by participants to introduce noise into the analysis that would bias towards the null. Despite this potential, we still observe statistically significant differences in the risk of transmission to household contacts depending on COVID-19 test type taken by the index case. We include this point in the limitations, on line 278-279:

> Sixth, it is possible that high-analytical-sensitivity tests could have turnaround times which we classify as rapid. However, such misclassification would bias toward the null.

3. The authors described that the study participants collected not only nasal swabs. Were these specimens for rapid testing or RT-PCR? Include a statement in the methods to clarify this.
Was the oropharyngeal specimen self-collected?

We appreciate the opportunity to clarify these points for readers. All specimens collected by participants in the study were self-collected at home, including oropharyngeal swab specimens. Of 2207 self-collected, unobserved oropharyngeal swab specimens collected by participants in the study, 0 adverse events (e.g. severe gaggine, vomiting, bleeding, swab breakage, choking) were reported by participants. Self-collected specimens were transported to the laboratory to undergo a high-analytical-sensitivity RT-qPCR test. We have modified lines 95-97 to include the requested details:

> Self-collected specimens (saliva, anterior nares swabs, oropharyngeal swabs, **Fig 1A,B**) from participants at home underwent laboratory testing for SARS-CoV-2 infection by a high analytical sensitivity RT-qPCR test, as previously described (Supplementary Information).

4. What was the minimum age of children included in the study? 6 or 8 years old? The age on main manuscript is different of the supplementary material?

We thank the Reviewer for bringing this issue to our attention. The minimum age of children included in the study was 6 years old, and those aged 8 or older provided written confirmed consent. We have modified lines 85-86 of the manuscript to clarify this:

> The study was approved by the California Institute of Technology IRB (protocol #20-1026). Individuals aged 6 and older were eligible for participation. Participants aged 8 years and older provided written informed consent, and all minors additionally provided verbal assent accompanied by written parental permission.

5. Line 176, Fig2A, how the unenrolled participants defined. Why did you include the unenrolled participants?

We appreciate the Reviewer for raising this question. Basic data about the unenrolled participants were used to help determine household transmission status, as enrolled participants reported their infection status, as described in lines 111 (Figure 1 caption):

> *6. Information about unenrolled household members was reported by enrolled participants.*

And 89-90 (Methods):

> At the conclusion of their participation, participants were asked to complete another questionnaire to report any SARS-CoV-2 test results from outside of the study, updated infection status of each household member (including those unenrolled), and infection-control practices performed.

Line 176 has been updated to also clarify that basic data from unenrolled household contacts were reported by study participants:

> We analyzed data from 370 individuals (enrolled participants and unenrolled household contacts reported by participants) of which 85 were defined as household index cases.

Data provided by enrolled household index cases was used to assess the effect of covariables on the risk of transmission.

Referee: 2

This manuscript described the SARS-CoV-2 transmission within 85 households and compared the transmission rate between nasal-swab rapid test and "other test types" grouped into other 6 types (nasal and not rapid, nasopharyngeal rapid, nasopharyngeal and not rapid, oral rapid and oral not rapid).

While the author provided rationale and prior evidence to support carrying out these analyses, the title/conclusion do not match the data presented (other factors that may contribute to the observed data) and there are major queries to the analyses, particularly when combining Phase 1 and Phase 2 participants. Please see below:

We thank the Reviewer for their thorough review of our manuscript and for raising insightful queries that help clarify and strengthen our work.

Major comments/queries:

1. As mentioned by the authors, the type of samples (saliva, nasal, nasopharyngeal) and the type of test have varied sensitivities and specificities. Furthermore, the sample medium and some of the sample types collected in Phase 1 and 2 are different. Please provide information regarding the type of test (RT-qPCR, saliva rapid test), and do you see a similar results if you compare nasal vs saliva vs nasopharyngeal and/or RT-qPCR vs rapid test regardless of sample types, which gene the test detects)? Do you expect the sample medium to influence the test sensitivity?

We appreciate the Reviewer for their engagement with the manuscript and attention to this detail. The Reviewer is correct that during Phase 1 of the study, specimens were collected in Spectrum SDNA 1000 devices, while in Phase 2, specimens were collected in Zymo SafeCollect devices. However, the media in both of these devices is guanidinium-thiocyanate based and designed to inactivate SARS-CoV-2 while preventing RNA degradation. The extraction method in each phase varied slightly, though in both phases, extraction was bead-based on an automated platform that successfully removes the collection and lysis buffer. Both subsequent RT-qPCR assays detect the SARS-CoV-2 N gene with high analytical sensitivity; we independently assessed the limit of detection for these assays to be at or below 1,000 copies/mL.

Further, each participant collected multiple specimen types, and specimens longitudinally for several days, with testing of these specimens used to define infection status, our outcome of interest in this study, for each participant. We do not believe that differences in specimen collection of processing in each study phase would impact the determination of infection status. In addition, although oropharyngeal swabs were only collected in Phase 2, paired nasal swabs and saliva were collected longitudinally, twice-per-day by participants in Phase 1; the paired specimen types and repeat testing in Phase 1 make it unlikely that infected individuals would be missed because oropharyngeal swab specimens were not collected in that Phase.

The Methods section has been updated for clarification:

> Self-collected specimens (saliva, anterior nares swabs, oropharyngeal swabs, **Fig 1A,B**) from participants at home underwent laboratory testing for SARS-CoV-2 infection by a high analytical sensitivity RT-qPCR test validated to have a limit of detection at or below 1,000 copies/mL for all specimen types in the study, as previously described (Supplementary Information).

2. As acknowledged by the author, the variant type is an important factor in transmission. The author concluded that rapid nasal swab increased transmission, if you compare nasal-swab rapid test and "other test types" within the Phase 1 (ancestral strain) and within Phase 2 (omicron), do you see the same results?

We appreciate this point raised by the reviewer. As listed in Table S1, during Phase 1 of the study, very few participants utilized rapid nasal COVID-19 tests. This is likely because rapid antigen diagnostic tests for COVID-19 did not become widely available for use until the end of Phase 1 of our study, in 2021. The few individuals who utilized rapid nasal

COVID-19 tests during Phase 1 of our study would not be sufficiently powered to discern the difference that we observe in the full cohort. However, in our mutivariable model we control for the effect of viral variant on risk of transmission, and still observe significantly higher transmission among individuals exposed to an index case first identified by a rapid nasal COVID-19 test.

To address this point by the Reviewer, we conducted our analysis on the subset of participants in households infected with the Omicron variant. While the lower number of participants widened the confidence intervals on point measurements, use of rapid nasal swab tests by index cases remained significantly associated with increased risk of transmission to household contacts compared to other test types. We have added a sentence on line 213 describing this result:

> When the analysis was limited to participants in households infected with the Omicron variant (**S5 Table**), use of rapid nasal swab tests by index cases remained significantly associated with increased transmission to household contacts compared to other test types (aOR 15.89 95% CI 1.59.-158.41).

In addition, we have added a new Table S5 to the Supplemental Information which provides the full results of this analysis:

> **S5 Table. Results of Modeled Risk of Transmission to Household Contacts of Index Cases Infected with Omicron Variant.** Among 101 household contacts of index cases infected with the Omicron variant, counts (N) of enrolled individuals who did not become infected during enrollment (uninfected) or became infected after the index case (secondary case) are provided for each covariable. The adjusted secondary attack rate (aSAR) and adjusted odds ratio (aOR) point estimates with 95% confidence intervals from multivariable analysis are listed for each covariable. As all households were infected with the Omicron variant, viral variant was removed from the sufficient set of covariables (**Figure 1C**). Of note, for 21 household contacts the index case test type was unknown and excluded from analysis. Additionally, two of two household contacts who were unvaccinated were both infected, resulting in collinearity of the vaccination covariable with infection status and omission of these two individuals. Similarly, six of six household contacts over the age of 60 were uninfected, resulting in collinearity of this age category with infection status and omission of these six individuals. Further, five of five individuals self-identifying as Native American were uninfected, resulting in collinearity of this race category with infection status and omission of these five individuals. A total of 67 household contacts were analyzed in this multivariable model. **(A)** Data for four covariables. **(B)** Association between COVID-19 test type used to identify the household index case, and subsequent transmission to household contacts when controlling for covariables. *Vaccinated status is defined as having received at least one dose of a COVID-19 vaccine at least 7 days prior to enrollment.

(A)

Exposure	Uninfected N	Uninfected (%)	Secondary Case N	Secondary Case (%)	Multivariable Model aSAR	95% CI	Pairwise Comparison Test	aOR	95% CI	Wald Test
**Age Category**	**64**		**37**							
<10	4	6.3	3	8.1	50.4	14.6-86.2		1.89	0.09-41.59	P=0.09
10 to 60	54	84.4	34	91.9	37.4	24.4-50.3	NA	1.00	*(Reference)*	
>60	6	9.4	0	0.0						
**Race/Ethnicity**	**64**		**37**							
Asian/PI	15	23.4	9	24.3	38.3	14.1-62.5		1.37	0.15-12.44	P=0.78
Biracial	3	4.7	3	8.1	36.5	1.1-71.9		1.19	0.06-22.77	P=0.91
Black	4	6.3	3	8.1	66.1	26.5-100.0		5.71	0.15-214.26	P=0.35
Native American	5	7.8	0	0.0			NA			
Unknown	4	6.3	4	10.8	66.6	29.0-100.0		11.59	0.36-389.02	P=0.17
White Hispanic	10	15.6	5	13.5	44.3	0.0-46.9		0.29	0.02-5.00	P=0.39
White Non-Hispanic	23	35.9	13	35.1	33.0	14.5-51.6		1.00	*(Reference)*	
**Vaccination Status**	**64**		**37**							
Not Vaccinated	0	0.0	2	5.4			NA	1.00	*(Reference)*	
Vaccinated*	64	100.0	35	94.6	38.1	25.3-50.9				
**Household Size**	**64**		**37**							
≤4	31	48.4	13	35.1	28.6	11.9-45.4	P=0.19	1.00	*(Reference)*	
>4	33	51.6	24	64.9	45.7	26.5-64.9		3.82	0.49-29.62	P=0.20

(B)

**Index Case Diagnostic Test**	**52**		**28**							
Rapid Nasal Test	34	65.4	8	28.6	55.6	35.2-76.1	**P=0.003**	15.89	1.59-158.41	**P=0.02**
Other Test Type	18	34.6	20	71.4	16.7	2.8-30.5		1.00	*(Reference)*	

3. What is the rationale for grouping individuals aged 10-60 years in a group? Host response and transmission in children or young adolescents are different from adults. Do you see the same results if the participants are stratified into different age groups, e.g. <12 years, 12-18 years, 19-60 years and >60 years.

We thank the Reviewer for pointing out the potential confusion. We previously determined the age categories based on a metanalysis synthesizing findings from 14 studies about the impact of age on COVID-19 transmission. The metanalysis found that "Compared to younger/middle-aged adults, susceptibility to infection for children younger than 10 years is estimated to be significantly lower, while estimated susceptibility to infection in adults older than 60 years is higher." (https://doi.org/10.1093/infdis/jiaa691). We have added a citation to this paper to line 42 and line 156:

> Age of both the index case (first person in the household to become infected) and at-risk household contacts (who either remain uninfected or become infected secondary cases) has also been implicated in SARS-CoV-2 household-transmission patterns.[6,14-17]
>
> The model adjusted for a sufficient set of the following potentially confounding variables: household size,[10-12] age,[6,14-17] race/ethnicity,[11,13] and vaccination status.[18-22]

To verify that we observe the same results with an alternative age grouping, we performed a re-analysis using the age categories suggested by the Reviewer. Under these new age groupings, individuals >60 remain at significantly lower risk of infection compared to individuals 19 to 60 years old, though additional categories results in smaller group sizes that widen the confident intervals on estimates. With these suggested age groupings, we continued to observe that index case use of a rapid nasal test was significantly associated with increased risk of transmission, compared to other test types. We have provided the results of this re-analysis as a new table in the Supplemental Information:

> **S4 Table. Results of Modeled Risk of Transmission to Household Contacts with Alternative Age Grouping.** Counts (N) of enrolled individuals who did not become infected during enrollment (uninfected) or became infected

after the index case (secondary case) are provided for each alternative age category. The adjusted secondary attack rate (aSAR) and adjusted odds ratio (aOR) point estimates with 95% confidence intervals from multivariable analysis are listed for each covariable. The same analysis in **Fig. 2A** is performed with alternative age stratification.

Exposure	Uninfected N	Uninfected (%)	Secondary Case N	Secondary Case (%)	Multivariable Model aSAR	95% CI	Pairwise Comparison Test	aOR	95% CI	Wald Test	Likelihood Ratio Test
**Age Category**	**149**		**82**								
<12	24	16.1	16	19.5	40.0	24.7-55.4	NA	0.95	0.36-2.53	P=0.92	P=0.06
12 to 18	26	17.4	12	14.6	31.8	17.6-46.0		0.75	0.27-2.07	P=0.58	
19 to 60	86	57.7	53	64.6	37.4	29.0-45.9		1.00	*(Reference)*		
>60	13	8.7	1	1.2	9.8	0.0-26.9c		0.07	0.01-0.74	**P=0.03**	
**Index Case Diagnostic Test**	**134**		**71**								
Rapid Nasal Test	28	20.9	29	40.8	53.7	35.1-68.5	**P=0.003**	4.92	1.67-14.50	**P=0.004**	P=0.07
Other Test Type	106	79.1	42	59.2	27.1	19.4-34.8		1.00	*(Reference)*		

And we have added a reference to this new supplementary table in the caption of Table 2 on line 235:

Analysis by alternate age categories is shown in **S4 Table**.

4. Figure 3b- do you include infection control practices as a covariable for analysis?

We thank the Reviewer for raising this question. Vaccination was included as a potential confounder which we control for in our model. Other infection control practices, such as masking and social distancing, are not included as a confounders in our model because we expected that adherence or specific technique hold may vary widely among households. Instead, we use a two-level random intercept model which accounts for random effects at the household level, which could capture the differential impact of infection control practices. Our model controls for viral variant, household density, age, race/ethnicity, and (as mentioned) vaccination status. This is described in lines 154-158 (Methods):

> The model adjusted for a sufficient set of the following potentially confounding variables: household size,[10-12] age,[6,14-17] race/ethnicity,[11,13] and vaccination status.[18-22] We also accounted for infecting SARS-CoV-2 viral variant.[18-20,28,32,33] Observations with missing data were omitted from respective analyses.
>
> We used this model to assess the effect of household prevention practices and the COVID-19 test type used to first identify the household index case.

5. Line 73: "we aimed to investigate whether test analytical sensitivity and differences in viral-load patterns among different specimen types may have implications for household transmission" – there are no investigation of viral load and test analytical sensitivity- the authors may have had shown this in previous publication on a subset of participants, but cannot assume this the same for all participants in this analyses?

We appreciate the Reviewer for their thoughtful comment. Quantitative viral load data from the incidence of infection in paired specimen types is rare, but available data suggests that SARS-CoV-2 frequently presents later in nasal swabs than saliva or throat swabs. Prior work by our group has shown that in a cohort of 7 individuals with incident pre-Delta infections (https://doi.org/10.1128/jcm.01785-21) and 14 individuals with incident Omicron infections (https://doi.org/10.1093/pnasnexus/pgad033), 17 (81%) of these 21 individuals exhibited an earlier rise in SARS-CoV-2 viral load in saliva or oropharyngeal swabs, compared to anterior nares nasal swabs. A similar trend, of virus presenting first in throat swabs compared to nasal swabs, was also observed among 10 of 18 infected participants in a SARS-CoV-2 human challenge study (https://doi.org/10.1038/s41591-022-01780-9). We agree with the Reviewer's point that this

viral-load pattern cannot be assumed to be the same for all participants in this study, though the high frequency of individuals exhibiting this specific viral-load pattern provides a plausible mechanism for the finding that on the population level, use of rapid nasal COVID-19 tests by index cases were associated with greater transmission to household than other test types. We understand that the phrasing of this sentence may suggest to readers that this study itself includes an analysis of viral loads, which it does not, and thus we've modified lines 73-75 to clarify this:

> In this study, we investigated whether previously observed viral-load patterns that affect the clinical sensitivity of low analytical sensitivity rapid COVID-19 tests have implications for household transmission.

Minor comments:

1. Line 34. "Use of nasal-swab rapid COVID-19 tests for initial detection of infection and initiation of infection control may not limit transmission as well as other test types." – this applies to household contacts.

We thank the Reviewer for this suggestion. We agree that clarifying this pertains to household contacts better captures our conclusions. We have modified lines 35-36 to reflect this change:

> Use of nasal-swab rapid COVID-19 tests for initial detection of infection and initiation of infection control may not limit transmission to household contacts as well as other test types

2. Line 187-188- should be in discussion

We appreciate the Reviewer commenting on this point. We felt it important to contextualize in the Results section that our study is not the first to demonstrate increased transmissibility observed among individuals infected with the Omicron variant. However, we understand the Reviewer's point that a statement such as this is typically presented in the Discussion rather than Results, and so we have modified this sentence on line 187 to:

> We observed that infection with the Omicron variant was associated with greater transmission than ancestral variants.

A separate statement comparing the above mentioned result to findings from other studies remains in the Discussion section, on line 264:

> We also observed, as other epidemiological studies have,[8,19,20,32-34] that infection with the Omicron variant was associated with increased transmission compared with ancestral viral variants.

Editorial Comments:

1. Please ensure that your manuscript meets PLOS ONE's style requirements, including those for file naming. The PLOS ONE style templates can be found at https://journals.plos.org/plosone/s/file?id=wjVg/PLOSOne_formatting_sample_main_body.pdf and https://journals.plos.org/plosone/s/file?id=ba62/PLOSOne_formatting_sample_title_authors_affiliations.pdf

We thank the Editor for providing this comment, and these resources. We have amended the formatting of our manuscript to match the PLOS ONE style requirements, including for file naming.

2. Thank you for stating in your Funding Statement: "This study is based on research funded in part by the Bill & Melinda Gates Foundation (INV-023124). The findings and conclusions contained within are those of the authors and do not necessarily reflect positions or policies of the Bill & Melinda Gates Foundation. This study was also funded in part by grants from the Ronald and Maxine Linde Center for New Initiatives at the California Institute

of Technology (to RFI), a grant from the Jacobs Institute for Molecular Engineering for Medicine at the California Institute of Technology (to RFI), a DGSOM Geffen Fellowship at the University of California, Los Angeles (to AVW), and the John Stauffer Charitable Trust SURF Fellowship at the California Institute of Technology (to JJ)." Please provide an amended statement that declares *all* the funding or sources of support (whether external or internal to your organization) received during this study, as detailed online in our guide for authors at http://journals.plos.org/plosone/s/submit-now. Please also include the statement "There was no additional external funding received for this study." in your updated Funding Statement. Please include your amended Funding Statement within your cover letter. We will change the online submission form on your behalf.

Our amended Funding Statement should read:

This study is based on research funded in part by the Bill & Melinda Gates Foundation (INV-023124). The findings and conclusions contained within are those of the authors and do not necessarily reflect positions or policies of the Bill & Melinda Gates Foundation. This study was also funded in part by grants from the Ronald and Maxine Linde Center for New Initiatives at the California Institute of Technology (to RFI), a grant from the Jacobs Institute for Molecular Engineering for Medicine at the California Institute of Technology (to RFI), a DGSOM Geffen Fellowship at the University of California, Los Angeles (to AVW), and the John Stauffer Charitable Trust SURF Fellowship at the California Institute of Technology (to JJ). The funders had no role in study design, data collection and analysis, decision to publish, or preparation of the manuscript. There was no additional external funding received for this study."

3. Thank you for stating the following financial disclosure: "This study is based on research funded in part by the Bill & Melinda Gates Foundation (INV-023124). The findings and conclusions contained within are those of the authors and do not necessarily reflect positions or policies of the Bill & Melinda Gates Foundation. This study was also funded in part by grants from the Ronald and Maxine Linde Center for New Initiatives at the California Institute of Technology (to RFI), a grant from the Jacobs Institute for Molecular Engineering for Medicine at the California Institute of Technology (to RFI), a DGSOM Geffen Fellowship at the University of California, Los Angeles (to AVW), and the John Stauffer Charitable Trust SURF Fellowship at the California Institute of Technology (to JJ)." Please state what role the funders took in the study. If the funders had no role, please state: ""The funders had no role in study design, data collection and analysis, decision to publish, or preparation of the manuscript."" If this statement is not correct you must amend it as needed. Please include this amended Role of Funder statement in your cover letter; we will change the online submission form on your behalf.

As described above, in Editor comment 2, Our amended Funding Statement should read:

This study is based on research funded in part by the Bill & Melinda Gates Foundation (INV-023124). The findings and conclusions contained within are those of the authors and do not necessarily reflect positions or policies of the Bill & Melinda Gates Foundation. This study was also funded in part by grants from the Ronald and Maxine Linde Center for New Initiatives at the California Institute of Technology (to RFI), a grant from the Jacobs Institute for Molecular Engineering for Medicine at the California Institute of Technology (to RFI), a DGSOM Geffen Fellowship at the University of California, Los Angeles (to AVW), and the John Stauffer Charitable Trust SURF Fellowship at the California Institute of Technology (to JJ). The funders had no role in study design, data collection and analysis, decision to publish, or preparation of the manuscript. There was no additional external funding received for this study."

4. Thank you for stating the following in the Competing Interests section: "I have read the journal's policy and the authors of this manuscript have the following competing interests: R.F.I. is a cofounder, consultant, and a director and has stock ownership of Talis Biomedical Corp. All other co-authors report no competing interests." Please confirm that this does not alter your adherence to all PLOS ONE policies on sharing data and materials, by including the following statement: ""This does not alter our adherence to PLOS ONE policies on sharing data and materials." (as detailed online in our guide for authors http://journals.plos.org/plosone/s/competing-interests). If there are restrictions on sharing of data and/or materials, please state these. Please note that we cannot proceed with consideration of your article until this information has been declared. Please include your

updated Competing Interests statement in your cover letter; we will change the online submission form on your behalf.

Our amended Competing Interests Statement should read:

I have read the journal's policy and the authors of this manuscript have the following competing interests: R.F.I. is a cofounder, consultant, and a director and has stock ownership of Talis Biomedical Corp. This does not alter our adherence to PLOS ONE policies on sharing data and materials. All other co-authors report no competing interests.

This change has been updated in lines 317-318 of the manuscript.

5. Please include captions for your Supporting Information files at the end of your manuscript, and update any in-text citations to match accordingly. Please see our Supporting Information guidelines for more information: http://journals.plos.org/plosone/s/supporting-information.

The following lines have been added to the Supporting Information section, which has been moved to immediately follow the main text of our manuscript, at line 302:

Supporting Information

S1 Table. Participant Demographics by (A) Study Phase and (B) Infecting SARS-CoV-2 Variant.

S2 Table. Univariable Model.

S3 Table. Association of Test Type Subcategories with SARS-CoV-2 Transmission Among Household Contacts.

S4 Table. Results of Modeled Risk of Transmission to Household Contacts with Alternative Age Grouping.

S5 Table. Results of Modeled Risk of Transmission to Household Contacts of Index Cases Infected with Omicron Variant.

Supplemental Methods

Supplemental References

Detailed Author Contribution Statements

These added captions can hyperlink to the Supplemental Information file provided.
