# Supplementary material for: Index cases first identified by nasal-swab rapid COVID-19 tests had more transmission to household contacts than cases identified by other test types
Source: PLoS One. 2023 Oct 5;18(10):e0292389. doi: 10.1371/journal.pone.0292389 (PMC10553276; doi:10.1371/journal.pone.0292389)
Supplement: Attachment — Submitted filename: PONE-D-23-09614_response_to_reviewers.pdf [file pone.0292389.s003.pdf]

Point-By-Point Response

Referee: 1

Regarding the limited sample size to conduct the analysis for Delta variant of SARS-CoV-2, the authors should state this as study limitation. It is important because the authors referred that both delta and omicron variants were circulating.

We thank the Reviewer for their comment. We do not present analyses stratified by Delta variant infections in the manuscript, because that analysis would be underpowered. To clarify for readers why Delta variant analyses are not presented, we have added the following sentence to line 159-162:

> We also accounted for infecting SARS-CoV-2 viral variant, [19-21, 29, 33, 34] either Omicron variant or ancestral variants. The study's second enrollment period occurred during increasing Omicron variant predominance. Thus, few participants in this study had Delta variant exposure, and analyses stratified by Delta variant infection would be insufficiently powered.

Referee: 2

Thank you for addressing the queries and appreciate the sub-analysis. I only have one minor comment in the abstract and discussion:

- line 35 and line 299: nasal-swab rapid test is less effective than other test, but that does not mean it does not limit transmission- please consider rewording to e.g. less effective than other test or something equivalent rather than 'no use' at all.

We thank the Reviewer once again for the thorough review of our manuscript and the follow-up comment. Lines 35 has been reworded accordingly.

> Use of nasal-swab rapid COVID-19 tests for initial detection of infection and initiation of infection control may be less effective at limiting transmission to household contacts than other test types.

Similarly, line 299 has also been reworded per the suggestion:

> However, results from this observational study suggest that the use of nasal-swab rapid COVID-19 tests to first identify infection does not limit household transmission as effectively as other test types.
